# Rare Neurologic Disease-Associated Mutations of AIMP1 Are Related with Inhibitory Neuronal Differentiation Which Is Reversed by Ibuprofen

**DOI:** 10.3390/medicines7050025

**Published:** 2020-05-06

**Authors:** Yu Takeuchi, Marina Tanaka, Nanako Okura, Yasuyuki Fukui, Ko Noguchi, Yoshihiro Hayashi, Tomohiro Torii, Hiroaki Ooizumi, Katsuya Ohbuchi, Kazushige Mizoguchi, Yuki Miyamoto, Junji Yamauchi

**Affiliations:** 1 Laboratory of Molecular Neuroscience and Neurology, Tokyo University of Pharmacy and Life Sciences, Hachioji, Tokyo 192-0392, Japan; takeuchi_yu@icloud.com (Y.T.); s169073@toyaku.ac.jp (M.T.); naaako_yu.052075@i.softbank.jp (N.O.); fy.yasu.823@docomo.ne.jp (Y.F.); miyamoto-y@ncchd.go.jp (Y.M.); 2 Laboratory of Applied Ecology, Tokyo University of Pharmacy and Life Sciences, Hachioji, Tokyo 192-0392, Japan; knoguchi@toyaku.ac.jp; 3 Laboratory of Oncology, Tokyo University of Pharmacy and Life Sciences, Hachioji, Tokyo 192-0392, Japan; yshrhys@toyaku.ac.jp; 4 Laboratory of Ion Channel Pathophysiology, Doshisha University, Kyotanabe, Kyoto 610-0394, Japan; ttorii@mail.doshisha.ac.jp; 5 Tsumura Research Laboratories, Tsumura & Co., Inashiki, Ibaraki 200-1192, Japan; ooizumi_hiroaki@mail.tsumura.co.jp (H.O.); oobuchi_katsuya@mail.tsumura.co.jp (K.O.); mizoguchi_kazushige@mail.tsumura.co.jp (K.M.); 6 Laboratory of Molecular Pharmacology, National Research Institute for Child Health and Development, Setagaya, Tokyo 157-8535, Japan

**Keywords:** AIMP1, mutation, differentiation, actin, ibuprofen

## Abstract

**Background:** Hypomyelinating leukodystrophy 3 (HLD3), previously characterized as a congenital diseases associated with oligodendrocyte myelination, is increasingly regarded as primarily affecting neuronal cells. **Methods:** We used N1E-115 cells as the neuronal cell model to investigate whether HLD3-associated mutant proteins of cytoplasmic aminoacyl-tRNA synthase complex-interacting multifunctional protein 1 (AIMP1) aggregate in organelles and affect neuronal differentiation. **Results:** 292CA frame-shift type mutant proteins harboring a two-base (CA) deletion at the 292th nucleotide are mainly localized in the lysosome where they form aggregates. Similar results are observed in mutant proteins harboring the Gln39-to-Ter (Q39X) mutation. Interestingly, the frame-shift mutant-specific peptide specifically interacts with actin to block actin fiber formation. The presence of actin with 292CA mutant proteins, but not with wild type or Q39X ones, in the lysosome is detectable by immunoprecipitation of the lysosome. Furthermore, expression of 292CA or Q39X mutants in cells inhibits neuronal differentiation. Treatment with ibuprofen reverses mutant-mediated inhibitory differentiation as well as the localization in the lysosome. **Conclusions:** These results not only explain the cell pathological mechanisms inhibiting phenotype differentiation in cells expressing HLD3-associated mutants but also identify the first chemical that restores such cells in vitro.

## 1. Introduction

In the central nervous system (CNS), oligodendrocytes often enlarge their plasma membranes by 100 times to wrap around the neuronal axons with multiple layers of myelin sheaths [1,2,3,4]. Their dynamic morphological changes involve the interactions of ligands with the cognate receptors such as cell adhesion molecular ones and also various signaling molecules, which all are cooperatively and strictly regulated during developmental stages. Myelin sheaths contribute to an indispensable role in the propagation of saltatory conduction in human and mammals. They also protect neuronal axons from physical and physiological stresses [1,2,3,4].

Hypomyelinating leukodystrophies (HLDs) are a group of recently classified hereditary neuropathies primarily impacting oligodendrocytes [5,6,7,8]. HLDs are reported to affect 1 in 200,000 to 500,000 people. HLD1, also called Pelizaeus-Merzbacher disease (PMD), is associated with various types of mutations (point mutations, deletion, and multiplication) of the *plp1* gene (OMIN ID. 312080). The gene product is the major myelin structural tetraspan-type membrane protein [4,5,6,7,8]. HLD2 (OMIN ID. 608803) is characterized by mutations in the *gjc2* gene (also called gja12), which encodes a tetraspan-type membrane protein found in gap junctions [9]. It is thought that these responsible gene products are required for generating and maintaining multiple layers of myelin sheaths and myelin homeostasis [5,6,7,8,9].

Advanced technologies involving nucleotide sequencing have enabled us to identify some unexpected disease-associated genes. Autosomal recessive HLD3 is associated with mutations in the *aminoacyl-tRNA synthetase complex-interacting multifunctional protein 1 (aimp1)* gene [10]: specifically, a frame-shift-type two-base deletion (C and A) at the 292nd position [292CA] and a nonsense-type mutation of Gln39-to-Ter [Q39X]) (OMIN ID. 260600). The gene product contributes to the multiple tRNA synthetase complex and probably also modulates the aminoacylation activities of tRNA synthetases [11]. HLD3 is characterized by early infantile onset of neuronal developmental delay with decreased myelination in the CNS as well as by peripheral spasticity [10]; recently, however, HLD3 has been suggested to affect neuronal cells primarily [12,13].

Like oligodendrocytes, neuronal cells undergo dynamic morphological changes during development, which involve regulated changes in the activities of many signaling and cytoskeletal molecules [14,15,16,17]. Here, we show that disease-associated mutant proteins (of the 292CA or Q39X types) of AIMP1 are primarily localized in the lysosome. Expression of the respective mutant proteins inhibits the elongation process in N1E-115 cells as the neuronal model reveals. Of note, the disease-specific peptide generated by the 292CA frame-shift-type mutation binds to β actin (simply called actin), which is partially colocalized with 292CA proteins with disorganized actin networks. 292CA and actin proteins can also be observed in the lysosome using lysosome immunoprecipitation [18]. Treatment with ibuprofen, a non-steroidal anti-inflammatory drug (NSAID) [19,20,21], reverses the inhibitory differentiation into phenotypes that is induced by AIMP1 mutations, ameliorating the molecular and cellular aberrances that are triggered by HLD3-associated AIMP1 mutant proteins in vitro.

## 2. Materials and Methods

### 2.1. Primary and Secondary Antibodies

The following antibodies were purchased: mouse monoclonal anti-endoplasmic reticulum (ER)-resident Grp78′s KDEL antigen (Cat. No. M181-3; immunofluorescence [IF], 1/200; immunoblotting [IB], 1/1000), mouse monoclonal anti-actin (for the β type; Cat. No. M177-3; IB, 1/20,000), mouse monoclonal anti-FLAG/DDDDK (Cat. No. M185-3; IB, 1/100,000), mouse monoclonal anti-GFP (Cat. No. M048-3; IB, 1/1,000; immunoprecipitation [IP], 0.25 μg/500 μg of proteins), and anti-goat rabbit or mouse IgG F(ab’) conjugated with horseradish peroxidase (Cat. Nos. 458 or 330; as secondary antibodies of IB, 1/5000) from MBL (Aichi, Japan); EasyBlot anti-rabbit- or mouse-specific IgG conjugated with horseradish peroxidase (Cat. Nos. GTX225856-01 or GTX225857-01; as secondary antibodies of IB following IP, 1/5,000) from GeneTex (Alton Pkwy Irvine, CA, USA); anti-actin (for the β type; Cat. No. 66009-1-1G; IF, 1/1,000) from Proteintech (Rosemont, IL, USA); mouse monoclonal anti-GM130 (Cat. No. 610822; IF, 1/200; IB, 1/100) from BD Biosciences (Franklin Lakes, NJ, USA); mouse monoclonal anti-lysosomal-associated membrane protein 1 (LAMP1) (Cat. No. ab25630; IF, 1/50; IB, 1/50) and rabbit polyclonal anti-SLC38A9 (Cat. No. ab130398; IP, 1 μg/500 μg of proteins; IB, 1/500) from Abcam (Cambridgeshire, UK); rabbit polyclonal growth-associated protein 43 (GAP43, Cat. No. 8945; IB, 1/500) from Cell Signaling Technology (Danvers, MA, USA); and goat anti-rabbit or mouse IgG conjugated with Alexa Fluor 488 or 594 (Cat. Nos. A11008, A11001, A11012, or A11005; as secondary antibodies of IF, 1/500) from Thermo Fisher Scientific (Waltham, MA, USA).

### 2.2. Plasmid Constructions

The nucleotides encoding human full-length AIMP1 (GenBank Acc. No. NM_004757) were synthesized by Fujifilm (Tokyo, Japan). The nucleotides encoding the frame-shift type (called 292CA) of two-base deletion (C and A) at the 292nd position and the nonsense type (called Q39X) of Gln-39 of AIMP1 (OMIN ID. 260600), as well as the construct encoding the 292CA-specific peptide (SGVYSSNNR IFWYQRTDKRRNRRRKESEREN), were also synthesized by Fujifilm. The 292CA-specific peptide sequence is not present in the amino acid sequence of the wild type AIMP1. These constructs were inserted into a pEGFP-C1 vector tagged with an enhanced variant of *A. victoria* GFP at the N-terminus or a pCMV5-FLAG-GFP vector tagged with FLAG and GFP in tandem at the N-terminus. The pEGFP-C1 and pCMV5-FLAG-GFP vectors were kindly provided by Dr. A. Tanoue (National Research Institute for Child Health and Development, Tokyo, Japan). All DNA sequences were confirmed through sequencing (Fasmac, Kanagawa, Japan).

### 2.3. Cell Culture, Differentiation, Plasmid Transfection, and Isolation of Stable Clones

The human 293T cell line, which exhibits a neuronal cell-like spindle-type morphology [15], was cultured on cell culture dishes (Greiner, Oberösterreich, Germany) in Dulbecco’s modified Eagle medium (DMEM, Thermo Fisher Scientific) containing 10% heat-inactivated FBS (GE Healthcare, Chicago, IL, USA) and PenStrep (Thermo Fisher Scientific) in 5% CO_2_ at 37 °C. 293T cells were purchased from Takara Bio (Shiga, Japan).

Mouse N1E-115 cells were cultured on cell culture dishes in DMEM/nutrient mixture F-12 (F-12, Thermo Fisher Scientific) containing 10% heat-inactivated FBS and PenStrep in 5% CO_2_ at 37 °C. To induce differentiation, N1E-115 cells were cultured on cell culture dishes with advanced TC polymer modification (Greiner) in culture medium without FBS in 5% CO_2_ at 37 °C for 2 or 3 days. Cells with a neurite-like process at least one cell body in length were considered to be differentiated. Cells had achieved more than 80% phenotype differentiation within three days following the induction of differentiation [16,17]. N1E-115 cells were kindly provided by Dr. D. Shiokawa (Tokyo University of Science, Chiba, Japan).

Cells were transfected with the respective plasmids using a ScreenFect A or ScreenFect A Plus transfection kit (FujiFilm) in accordance with the manufacturer’s instructions. The medium was generally replaced 4 h after transfection and used for experiments every 44 h thereafter. Attached, trypan-blue-incorporating cells were less than 5% in each experiment.

For the collection of N1E-115 cells stably harboring the wild type or the mutant construct of AIMP1, cells were transfected with the plasmid encoding the respective constructs in a 3.5 cm cell culture dish. Growth medium containing 500 μg/mL G418 (Nacalai Tesque, Kyoto, Japan) was changed every 2 or 3 days. After at least 14 days, G418-resistant colonies were collected. They were further cultured for another 14 days and then used for experiments. For experiments using stable clones, attached, trypan-blue-incorporating cells were also less than 5% in each experiment.

### 2.4. Fluorescence Images

Cells on a coverslip were fixed with 4% paraformaldehyde (Nacalai Tesque) or 100% cold methanol (Nacalai Tesque) as described elsewhere [17]. Cells were blocked with Blocking One reagent (Nacalai Tesque) and incubated first with a primary antibody and then with an Alexa Fluor-conjugated secondary antibody (Thermo Fisher Scientifi). The coverslips on the slide glass were mounted with Vectashield reagent (Vector Laboratories, Burlingame, CA, USA). The TIFF images were collected with a microscope system equipped with a laser-scanning Fluoview apparatus (FV1000D and FV1200, Olympus, Tokyo, Japan) using Fluoview software (Olympus). The resulting colored images were analyzed in Image J software (URL: https://imagej.nih.gov/) for line plots. Each of the images in the figures is representative of three experimental results.

### 2.5. Non-Denatured and Denatured Polyacrylamide Gel Electrophoresis and Immunoblotting

Cells were lysed in buffer A (50 mM HEPES-NaOH, pH 7.5, 150 mM NaCl, 5 mM MgCl_2_, 1 mM phenylmethane sulfonylfluoride, 1 μg/mL leupeptin, 1 mM EDTA, 1 mM Na_3_VO_4_, 10 mM NaF, and 0.5% NP-40) as described elsewhere [16,17]. The samples of total cell lysates were mixed with sample buffer without 2-mercaptoethanol or mixed and boiled with sample buffer with 2-mercaptoethanol (Cat. Nos. 09500-6 or 09499-14, Nacalai Tesque) for non-denaturing or denaturing polyacrylamide gels (Cat. Nos. 13064-64 or 13070-74, Nacalai Tesque), respectively. The proteins were separated electrophoretically, then transferred to polyvinylidene difluoride membranes, blocked with Blocking One reagent, and immunoblotted using a primary antibody followed by a peroxidase-conjugated secondary antibody (MBL or GeneTex). The bound antibodies were chemiluminescently detected by exposing X-ray film (FujiFilm). The films were captured as TIFF image files using an Epson (Nagano, Japan) scanner and its driver software (Epson). The pixels for immunoreactive species were measured in the segment analysis mode using UN-SCAN-IT software (URL: https://www.silkscientific.com/gel-analysis.htm). Images in figures are representative of three experimental results.

### 2.6. Immunoprecipitation for the Purposed Protein or the Lysosome

Supernatants of the cell lysates in buffer A were used for immunoprecipitation of the purposed proteins as described elsewhere [16,17]. The supernatants were mixed with protein G resin (GE Healthcare, Fairfield Easton, CT, USA) that had been absorbed with an antibody. The immunoprecipitates in supernatants of the cell lysate were denatured, subjected to denaturing polyacrylamide gel electrophoresis, and blotted onto polyvinylidene difluoride membranes for immunoblotting.

For immunoprecipitation of the lysosome [18], we used buffer B (50 mM HEPES-NaOH, pH 7.5, 150 mM NaCl, 5 mM MgCl_2_, 1 mM phenylmethane sulfonylfluoride, 1 μg/mL leupeptin, 1 mM EDTA, 1 mM Na_3_VO_4_, 10 mM NaF) and homogenized cell lysates with Potter-Elvehjem homogenizer. The homogenates were centrifuged at 150× *g* for 10 min in a tabletop centrifuge. The supernatants were gently mixed with an anti-SLC38A9 antibody-absorbed protein G resin according to their respective manufacturer’s instructions. The immunoprecipitates were denatured, subjected to denaturing gel electrophoresis, and blotted for immunoblotting.

### 2.7. Mass Spectrometry of an Interacting Protein

293T cells (1 × 10^9^) were transiently transfected with pCMV5-FLAG-GFP (for control protein expression) or pCMV5-FLAG-GFP-292CA-specific peptide. After 48 h, cells were lysed in lysis buffer A. Protein complexes containing the transfected proteins were isolated from cleared supernatants with a FLAG-tagged Protein Mild Purification Kit (MBL) according to the manufacturer’s instruction. Fractions containing the complexes were filtered through a 3000-molecular-weight-cutoff membrane (Millipore, Billerica, MA, USA), subjected to denaturing polyacrylamide gel electrophoresis, and visualized by silver staining (Cosmo Bio, Tokyo, Japan). Immunoreactive species were excised and enzymatically digested in-gel (Cosmo Bio). The resultant peptides were analyzed with an Ettan MALDI-TOF Pro mass spectroscope (Cosmo Bio) and the data were processed using a Mascot database (URL: http://www.matrixscience.com/).

### 2.8. Statistical Analysis

Values are means ± SD from separate experiments. Intergroup comparisons were performed by means of Student’s t test using Microsoft (Redmond, WA, USA) Excel. For more than three samples, a one way analysis of variance (ANOVA) was followed by a Fisher’s protected least significant difference test as a post hoc comparison using AnalystSoft StatPlus (URL: https://www.analystsoft.com/jp/). Differences were considered significant when *p* < 0.05.

### 2.9. Ethics Statement

Gene recombination techniques in vitro and in vivo were performed in accordance with a protocol approved by both the Tokyo University of Pharmacy and Life Sciences Gene and Animal Care Committee and the Japanese National Research Institute for Child Health and Development Gene and Animal Care Committee. Ethical approval codes: L20-04 and L20-05, Date of approval: 1 April 2020.

## 3. Results

### 3.1. 292CA or Q39X Mutant Proteins of AIMP1 are Mainly Localized in the Lysosome

**Figure 1:**
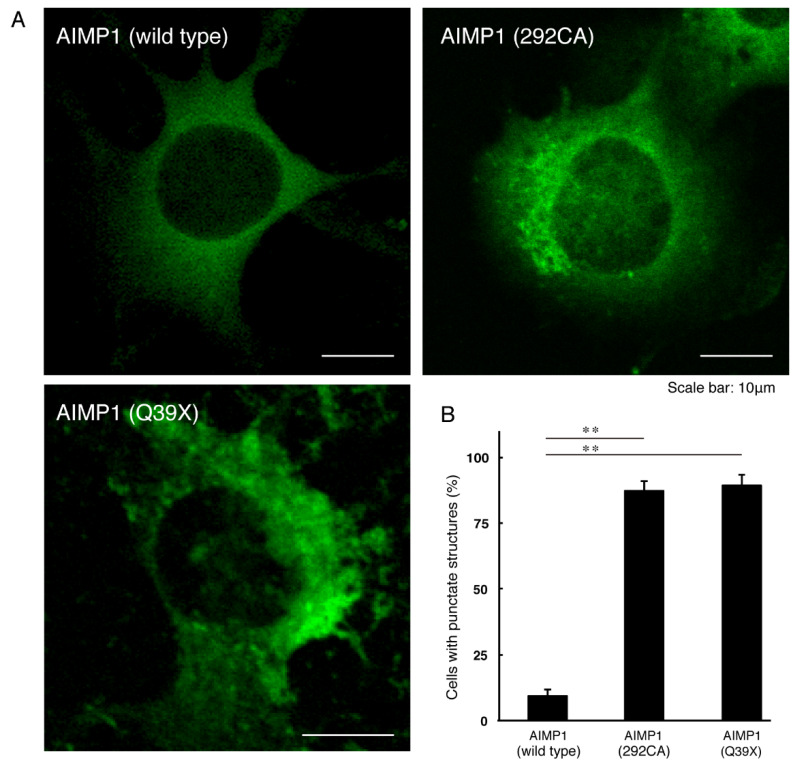
The 292CA or Q39X mutant proteins of AIMP1 are present in aggregate-like punctate structures whereas the wild type proteins are expressed in the cytoplasm. (**A**) Mouse N1E-115 cells were transfected with the plasmid encoding the wild type AIMP1 or the 292CA or Q39X mutant construct with a GFP-tag. Representative fluorescence images (green) are shown. The scale bars indicate 10 μm. (**B**) The percentages of cells with punctate structures are shown in a graph (**, *p* < 0.01; n = 3 fields [total 240 cells]).

To investigate whether the 292CA or Q39X mutation of cytoplasmic AIMP1 affects the subcellular localization of AIMP1, we transfected the plasmid encoding the GFP-tagged, wild type AIMP1 or AIMP1 encoding the 292CA or Q39X mutation into mouse neuronal cell line N1E-115. The wild type AIMP1 proteins were localized in the cytoplasm [10,11]; the 292CA or Q39X mutant proteins, in contrast, appeared as punctate structures throughout the cell bodies of approximately 80% of transfected cells (Figure 1A,B). This study focuses on genetic mutations in human. It could be appropriate to use a human cell line; but we used N1E-115 cells to determine how mutants affect cell differentiation. The first reson is that a human cell line such as SH-SY5Y does not display the typical differentiated phenotype with long neurite-like processes. Second, a human cell line is not effectively transfected using a normal transfection method such as lipofection. Third, the reproducibility of experiments in N1E-115 cells is good because the differentiation conditions of N1E-115 cells are not difficult.

**Figure 2:**
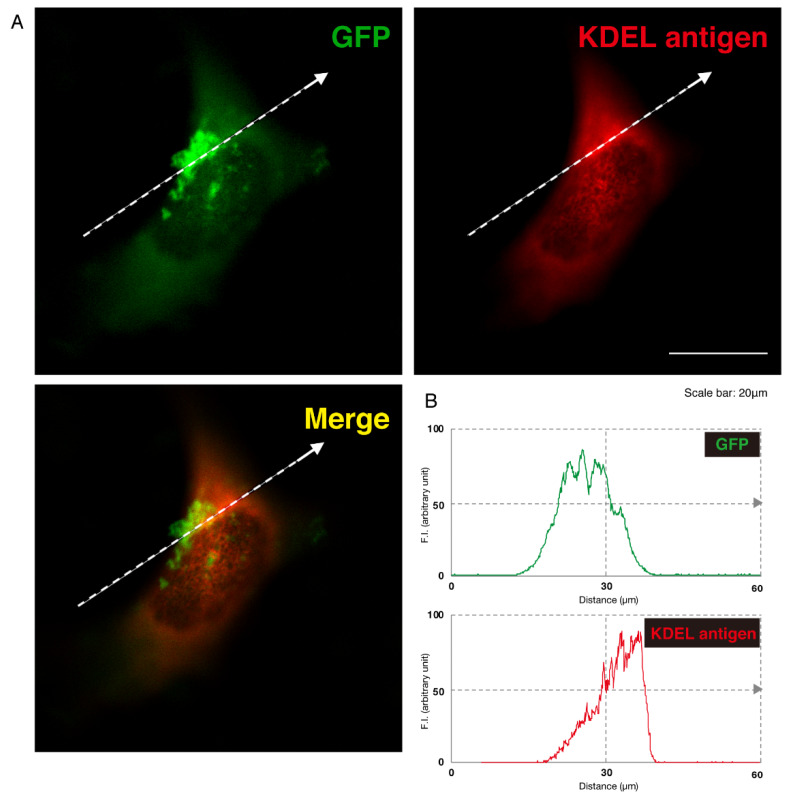
The 292CA mutant proteins of AIMP1 are not present in the endoplasmic reticulum (ER). (**A**) N1E-115 cells were transfected with the plasmid encoding the 292CA mutant construct of GFP-tagged AIMP1 (green). Transfected cells were stained with an antibody against the ER marker KDEL antigen (red). A merged image with a dotted arrow is also shown in the bottom panel. The scale bar indicates 20 μm. (**B**) Fluorescence intensities (F.I., arbitrary unit) of green and red colors are shown along dotted arrows in **A**.

**Figure 3:**
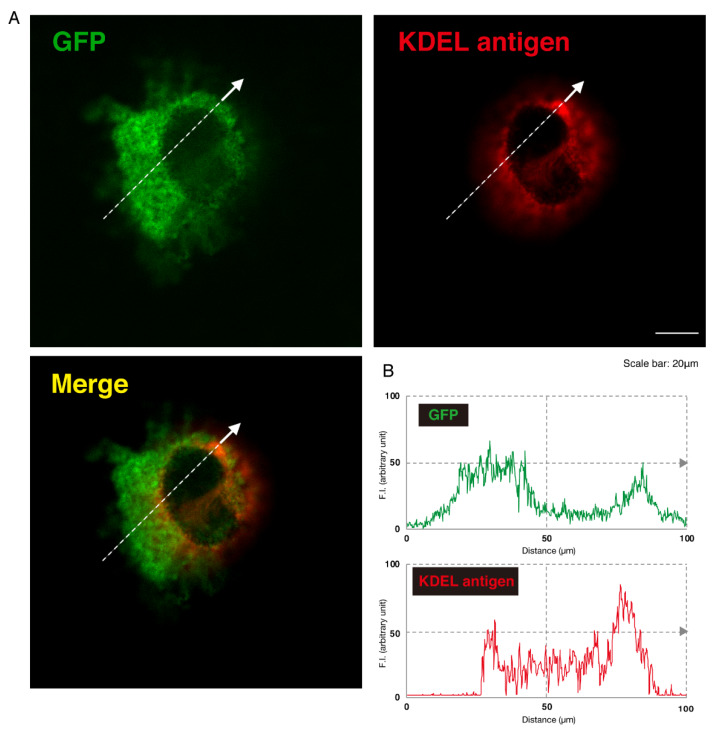
The Q39X mutant proteins are not present in the ER. (**A**) N1E-115 cells were transfected with the plasmid encoding the Q39X mutant construct of GFP-tagged AIMP1 (green). Transfected cells were stained with an antibody against the ER marker KDEL antigen (red). A merged image with a dotted arrow is also shown in the bottom panel. The scale bar indicates 20 μm. (**B**) Fluorescence intensities (F.I., arbitrary unit) of green and red colors are shown along dotted arrows in **A**.

**Figure 4:**
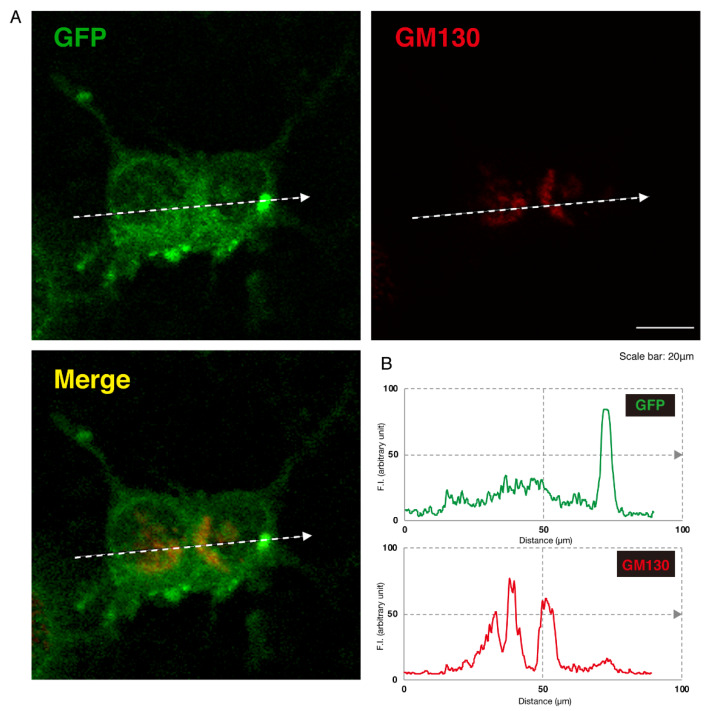
The 292CA mutant proteins are partially colocalized with the Golgi body marker. (**A**) N1E-115 cells were transfected with the plasmid encoding the 292CA mutant construct of GFP-tagged AIMP1 (green). Transfected cells were stained with an antibody against the Golgi body marker GM130 (red). A merged image with a dotted arrow is also shown in the bottom panel. The scale bar indicates 20 μm. (**B**) Fluorescence intensities (F.I., arbitrary unit) of green and red colors are shown along dotted arrows in **A**.

**Figure 5:**
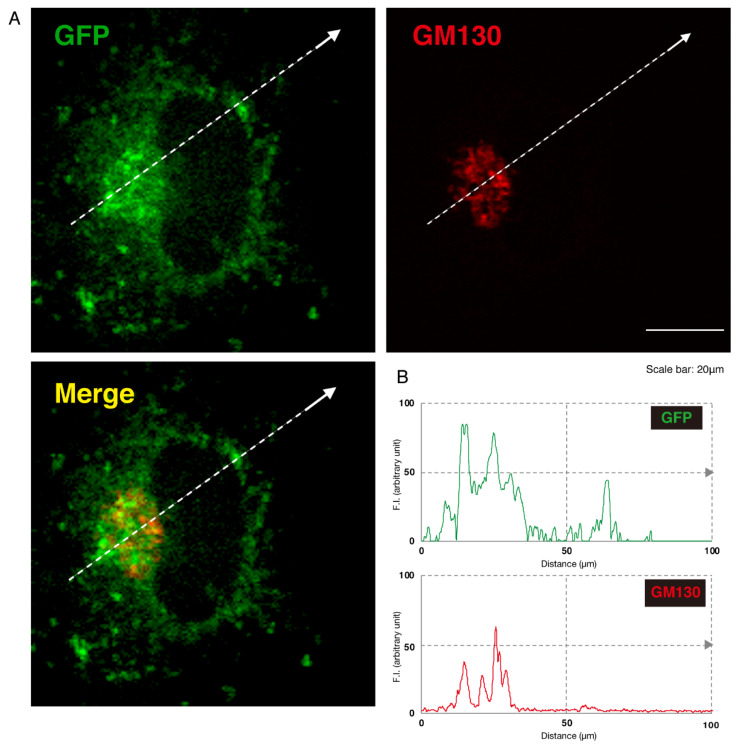
The Q39X mutant proteins are partially colocalized with the Golgi body marker. (**A**) N1E-115 cells were transfected with the plasmid encoding the Q39X mutant construct of GFP-tagged AIMP1 (green). Transfected cells were stained with an antibody against the Golgi body marker GM130 (red). A merged image with a dotted arrow is also shown in the bottom panel. The scale bar indicates 20 μm. (**B**) Fluorescence intensities (F.I., arbitrary unit) of green and red colors are shown along dotted arrows in **A**.

**Figure 6:**
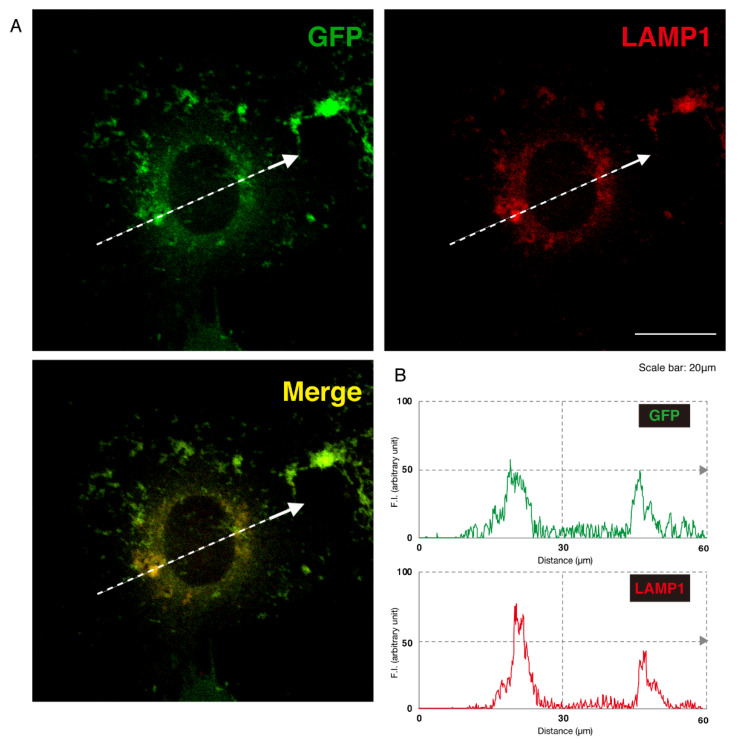
The 292CA mutant proteins are colocalized with the lysosome marker. (**A**) N1E-115 cells were transfected with the plasmid encoding the 292CA mutant construct of GFP-tagged AIMP1 (green). Transfected cells were stained with an antibody against the lysosome marker LAMP1 (red). A merged image with a dotted arrow is also shown in the bottom panel. The scale bar indicates 20 μm. (**B**) Fluorescence intensities (F.I., arbitrary unit) of green and red colors are shown along dotted arrows in **A**.

**Figure 7:**
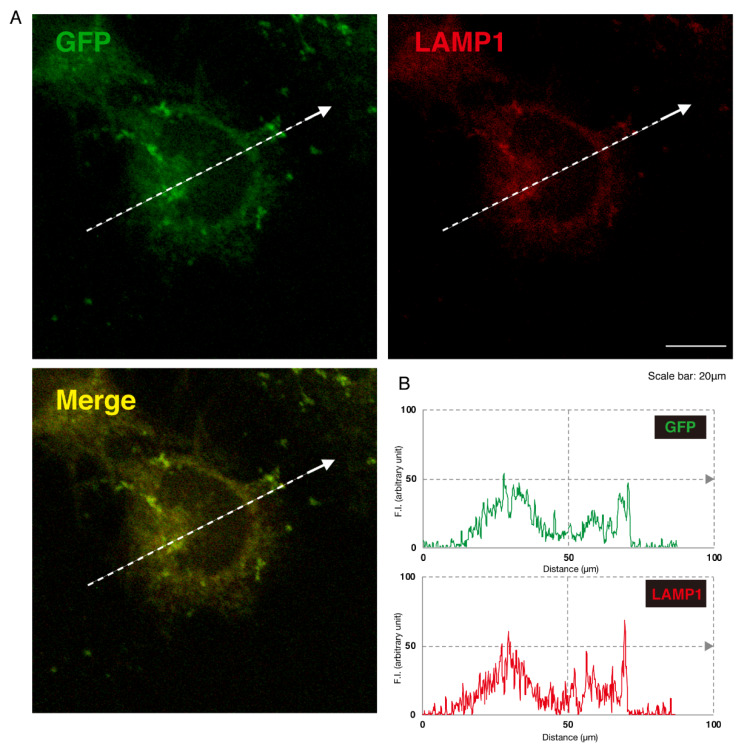
The Q39X mutant proteins are not present in the lysosome marker. (**A**) N1E-115 cells were transfected with the plasmid encoding the Q39X mutant construct of GFP-tagged AIMP1 (green). Transfected cells were stained with an antibody against the lysosome marker LAMP1 (red). A merged image with a dotted arrow is also shown in the bottom panel. The scale bar indicates 20 μm. (**B**) Fluorescence intensities (F.I., arbitrary unit) of green and red colors are shown along the dotted arrows in **A**.

Given this, we asked which organelles correspond to these punctate structures. First, we transfected the plasmid encoding the GFP-tagged, mutated AIMP1 (292CA or Q39X) into N1E-115 cells and stained cells with an antibody against the KDEL antigen, which specifically recognizes the endoplasmic reticulum (ER). The GFP-tagged 292CA or Q39X mutant proteins partially exhibited colocalization with the red fluorescence-stained KDEL antigen but the majority of their signals did not colocalize (Figure 2A,B and Figure 3A,B). Next, when we stained cells transfected with the plasmid encoding the GFP-tagged, mutated AIMP1 with an antibody against GM130 as a Golgi body-specific antigen, the 292CA and Q39X proteins exhibited partial merged localization with the red fluorescence-stained GM130 antigen (Figure 4A,B and Figure 5A,B). Then we introduced GFP-tagged, mutated AIMP1 into cells and analyzed its colocalization with the lysosome-specific, red fluorescence-stained lysosomal-associated membrane protein 1 (LAMP1) antigen (Figure 6A,B and Figure 7A,B). Both the 292CA and the Q39X proteins exhibited colocalization with LAMP1, indicating that the 292CA or Q39X proteins are localized not in the cytoplasm but rather in the lysosome and partially in the Golgi body as well as in the ER.

### 3.2. 292CA or Q39X Mutant Proteins Forms Oligomers

**Figure 8:**
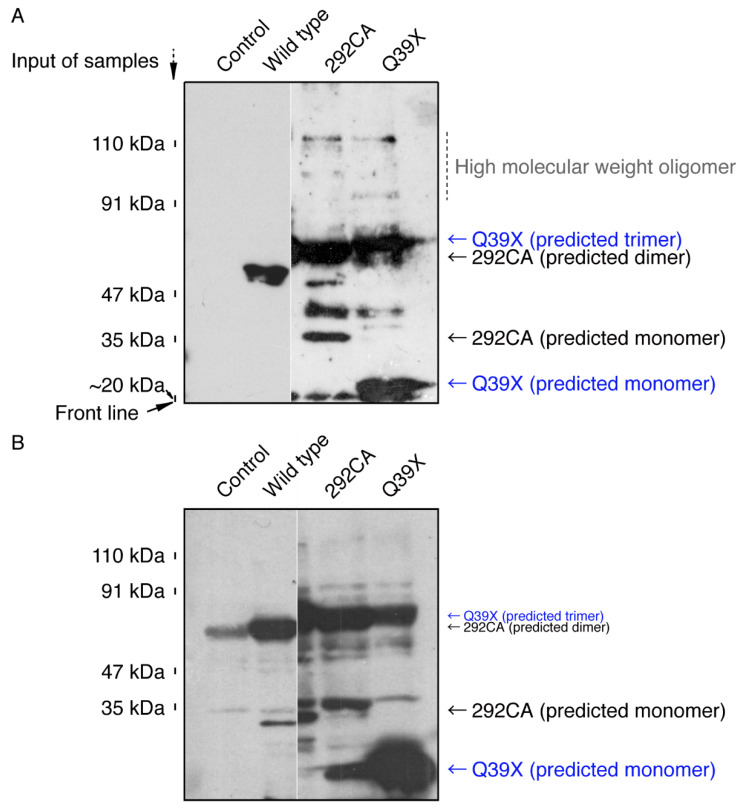
The 292CA or Q39X mutant proteins exhibit oligomeric forms in a non-denatured polyacrylamide gel. 293T cell lysates expressing the wild type AIMP1 proteins with GFP-tag or the 292CA or Q39X mutant proteins were subjected to a non-denaturing polyacrylamide gel (**A**) or a denaturing polyacrylamide gel (**B**). Blotted membranes were incubated with an anti-GFP antibody. In a non-denatured polyacrylamide gel, the wild type proteins mainly corresponded to a monomeric form whereas the mutant ones exhibited molecular weights characteristic of dimeric proteins at least. Aggregated proteins appear as thick as the electrophoresis lanes.

To estimate whether AIMP1 mutant proteins form protein aggregates that are localized in the lysosome, we subjected protein samples from cells transfected with the wild type or either of the mutants to native non-denaturing or normal denaturing polyacrylamide gel electrophoresis. In the native gel electrophoresis, the 292CA or Q39X mutant proteins exhibited probable dimeric or polymeric immunoreactive species (Figure 8A). Some predicted degradation immunoreactive species were also observed. Similar results were also obtained using normal gel electrophoresis (Figure 8B), suggesting that mutant proteins may be present as strongly aggregated forms.

### 3.3. 292CA Mutant Proteins Specifically Interact With Actin

**Figure 9:**
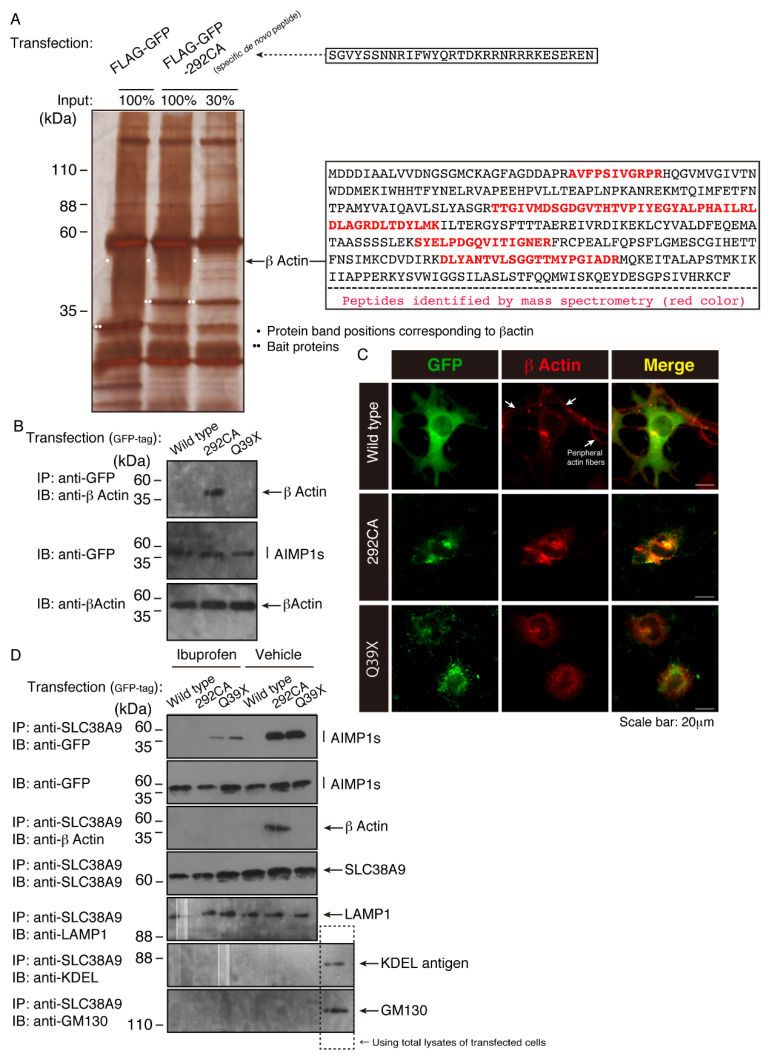
The 292CA mutant proteins interact with actin and treatment with ibuprofen decreases this interaction. (**A**) The total amounts (100%) or 30% of all immunoprecipitates from human 293T cell lysates expressing the control FLAG-GFP or the 292CA-specific de novo peptide with FLAG-GFP tag (right upper letters) were subjected to a denatured polyacrylamide gel and the gel was silver-stained (left image). The resultant peptides digested from an approximately 50-kDa protein specific to 292CA were analyzed with an Ettan MALDI-TOF Pro mass spectroscope and identified as human actin (β type actin, right bottom letters with red color [mass spectroscopically-identified peptides]). The single and double white circles indicate proteins corresponding to actin position and bait, respectively. (**B**) N1E-115 cells were transfected with the plasmid encoding the wild type construct or the 292CA or Q39X mutant of GFP-tagged AIMP1 and lysed. The lysates were immunoprecipitated (IP) with an anti-GFP antibody and immunoblotted (IB) with an anti-actin antibody (upper panel). The lysates were also immunoblotted with an antibody against GFP or actin (middle or bottom panel). (**C**) N1E-115 cells transfected with the respective GFP-tagged AIMP1 constructs (green) were immunostained with an anti-actin antibody (red). Merged images are also shown. The arrows indicate actin fibers in the cell periphery, which are seen only in the case of transfection with the wild type. The scale bars indicate 20 μm. (**D**) Lysates of transfected N1E-115 cells in culture with or without ibuprofen were immunoprecipitated with an anti-SLC38A9 antibody (for the lysosome) and immunoblotted with an antibody against GFP (for each of the AIMP1 constructs), actin, SLC39A9, LAMP1, KDEL antigen, or GM130. The immunoblots for GFP-tagged AIMP1 proteins (the wild type or each of the mutants), KDEL antigen, or GM130 for total lysates are shown.

Since the 292CA frame-shift-type mutant proteins uniquely generate de novo peptides, we tried to identify 292 CA de novo peptide-interacting protein(s) by means of affinity-precipitation in the lysate of the 293T human cell line, which has ectodermal properties as well as high protein expression levels [15]. Although many silver-stained proteins were observed in affinity-precipitation with control FLAG-tagged GFP proteins, an approximately 50 kDa protein was specifically observed in affinity-precipitation with FLAG and GFP-tagged 292CA de novo peptide. Through mass spectroscopy, we identified the 50 kDa protein as cytoplasmic actin (β type actin; Figure 9A).

Thus we transfected plasmids encoding the GFP-tagged wild type proteins or GFP-tagged 292 CA or Q39X proteins into N1E-115 cells and examined the interaction of endogenous actin with the wild type or the 292 CA or Q39X mutants by means of immunoprecipitation or immunofluorescence. The GFP-tagged 292CA proteins, but not the wild type or the Q39X proteins, were specifically immunoprecipitated with actin (Figure 9B). Consistently, the 292CA proteins were colocalized with actin proteins in disorganized actin cytoskeletal networks; in contrast, neither the wild type nor the Q39X proteins were colocalized with them (Figure 9C). In cells transfected with the wild type, peripheral actin fibers along neurite-like processes were clearly observed. Though the Q39X proteins did not interact with actin, they exhibited disorganized actin networks. The reason may be due to that presence of the Q35X protein aggregates inhibits actin fiber formation. Alternatively, the Q35X proteins may interact with molecule(s) belonging to actin protein networks to disturb actin fiber formation. In either case, it is likely that AIMP1 mutations are associated with inhibiting the formation of actin fibers.

We next asked whether the 292CA mutant proteins occur together with actin in the lysosome fraction. We confirmed that immunoprecipitates (organelle precipitates) with an antibody against SLC38A9 [18], which is specifically expressed in the lysosome, contained 292CA or Q35X mutant proteins in the respective transfected cells (Figure 9D and Figure S1). In contrast, the wild type was not present in immunoprecipitates with an anti-SLC38A9 antibody. Also, we specifically detected actin proteins in immunoprecipitates from cells transfected with 292CA, but not in those from cells transfected with Q35X, suggesting that 292CA mutant proteins are present with actin in the lysosome. Immunoprecipitates with an anti-SLC38A9 antibody definitely contained antigens for the LAMP1 antigen but not for the KDEL or GM130 antigens.

### 3.4. Cells Harboring the 292CA or Q39X Mutant Constructs Fail to Exhibit Differentiated Phenotypes

**Figure 10:**
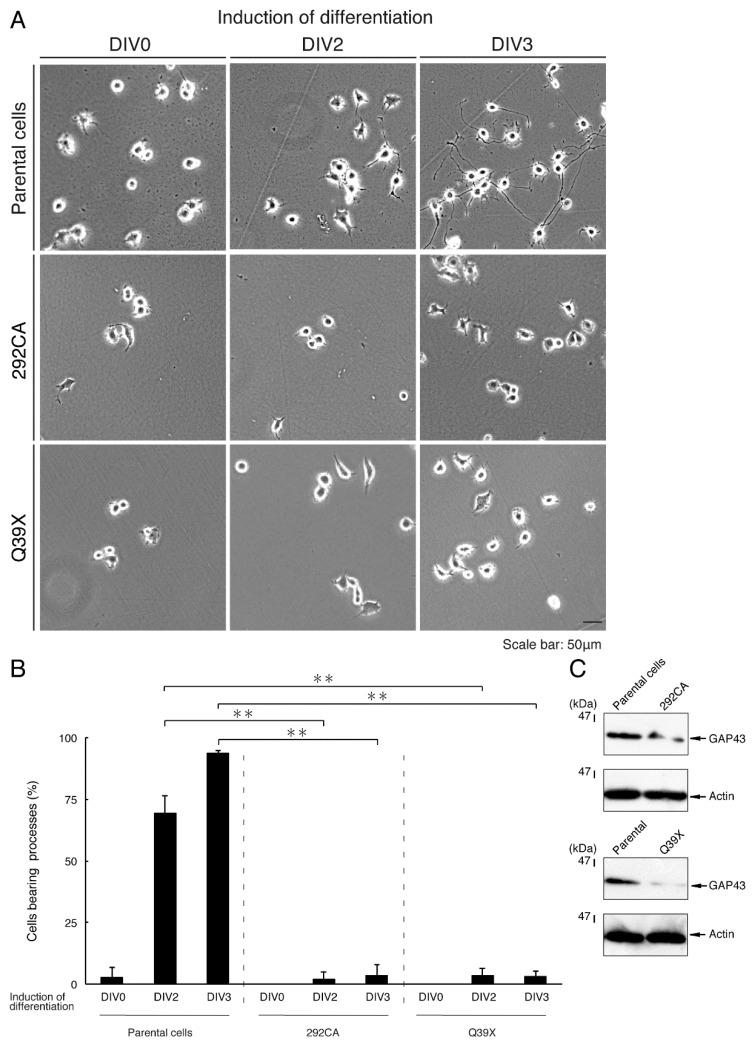
Cells harboring each of the AIMP1 mutant constructs fail to exhibit differentiated phenotypes. (**A**,**B**) We allowed N1E-115 cells stably harboring the 292CA or Q39X mutant constructs of AIMP1 or parental cells to differentiate for 0, 2, or 3 days (DIV0, DIV2, or DIV3). The scale bar indicates 50 μm. Percentages of differentiated cells bearing neurite-like processes that were at least one cell body in length are statistically shown (**, *p* < 0.01; n = 3 fields [total 240 cells]). (**C**) On day 3 following the induction of differentiation, cells were lysed and immunoblotted with an anti-GAP43 or actin antibody.

Given that the 292CA or Q39X mutation changed the properties of AIMP1 proteins, including their intracellular localization, we explored whether either of these mutants could affect neuronal differentiation. We generated N1E-115 cells stably harboring the 292CA or Q39X mutant constructs through drug-resistant screening and allowed cells to differentiate. Following the induction of differentiation, parental cells triggered neuronal differentiation programs that stimulated outgrowth, and the extended neurite-like processes formed cell bodies [16,17]. 2 or 3 days following the induction of differentiation, almost all cells exhibited processes that were at least one cell body in length (Figure 10A,B). On the other hand, cells harboring the 292CA or Q39X mutant constructs did not extend processes sufficiently. These results were consistent with those from an immunoblotting experiment using an antibody against growth-associated protein 43 (GAP43), which is the specific neuronal differentiation marker (Figure 10C); the degree of immunoblotting for an antibody to control actin was comparable between parental cells and cells harboring each of the respective mutants.

**Figure 11:**
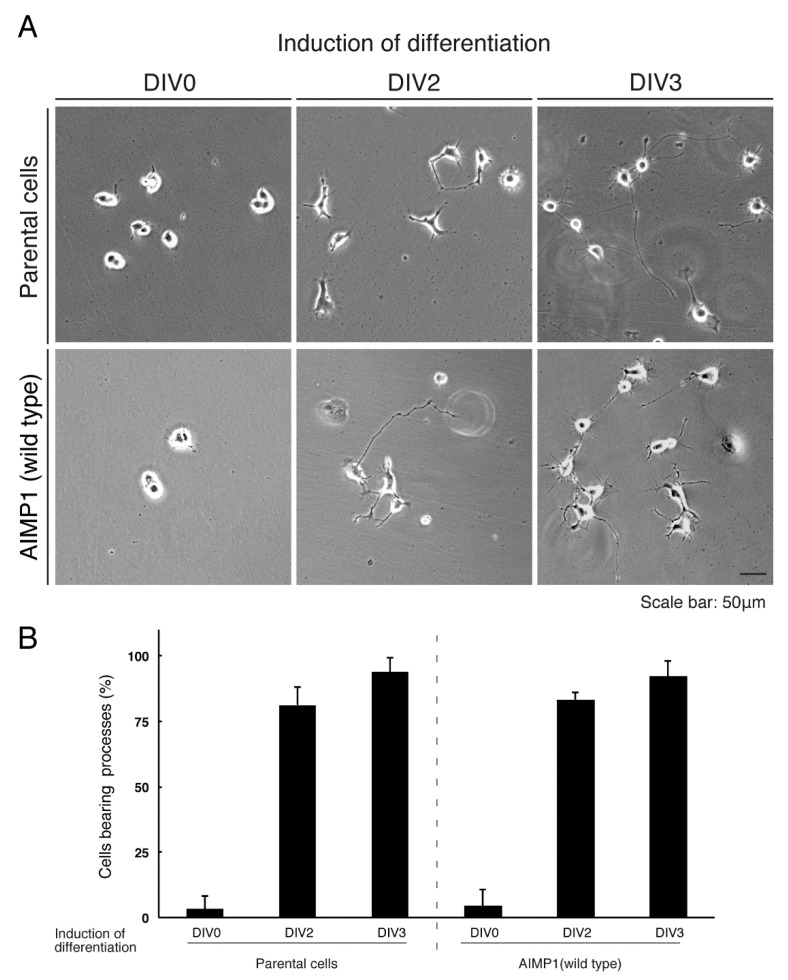
Cells harboring the wild type AIMP1 exhibit differentiated phenotypes. (**A**,**B**) We allowed N1E-115 cells stably harboring the wild type AIMP1 construct or parental cells to differentiate for 0, 2, or 3 days (DIV0, DIV2, or DIV3). The scale bar indicates 50 μm. Percentages of differentiated cells bearing neurite-like processes that were at least one cell body in length are statistically shown (n = 3 fields [total 240 cells]).

To confirm that parental N1E-115 cells undergo differentiation similar to that seen in cells stably harboring wild type AIMP1, we isolated the cells through drug-resistant screening. The differentiation efficiencies of the parental cells were comparable to those of cells harboring the wild type constructs (Figure 11A,B).

Taking these findings together with the results described above, we concluded that N1E-115 cells harboring 292 CA or Q35X mutant constructs exhibited undifferentiated phenotypes with disorganized actin fibers, whereas parental cells or cells harboring the wild type exhibited differentiated phenotypes with normal peripheral actin fibers.

### 3.5. Ibuprofen Reverses Inhibitory Differentiation of Phenotypes Among Cells Harboring the 292CA or Q39X Mutant Constructs

**Figure 12:**
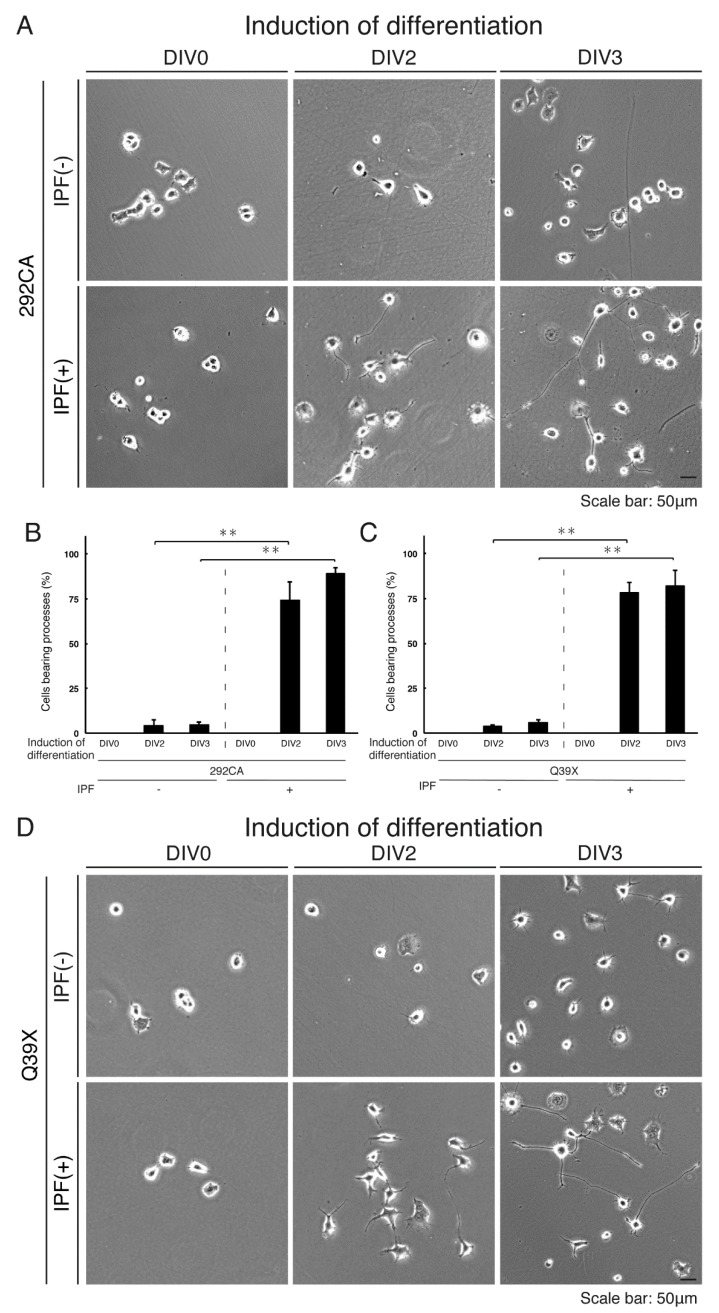
Treatment with ibuprofen reverses phenotypes of cells harboring each of the AIMP1 mutant constructs. (**A**,**B**) We allowed N1E-115 cells stably harboring the 292CA mutant constructs to differentiate for 0, 2, or 3 days (DIV0, DIV2, or DIV3) in the presence or absence (vehicle control) of ibuprofen (IPF, 100 μM). Percentages of differentiated cells bearing neurite-like processes that were at least one cell body in length are statistically shown (**, *p* < 0.01; n = 3 fields [total 240 cells]). The bar indicates 50 μm. (**C**,**D**) We allowed N1E-115 cells stably harboring the Q39X mutant constructs to differentiate for 0, 2, or 3 days (DIV0, DIV2, or DIV3). Percentages of differentiated cells bearing neurite-like processes that were at least one cell body in length are statistically shown (**, *p* < 0.01; n = 3 fields [total 240 cells]). The bar indicates 50 μm.

**Figure 13:**
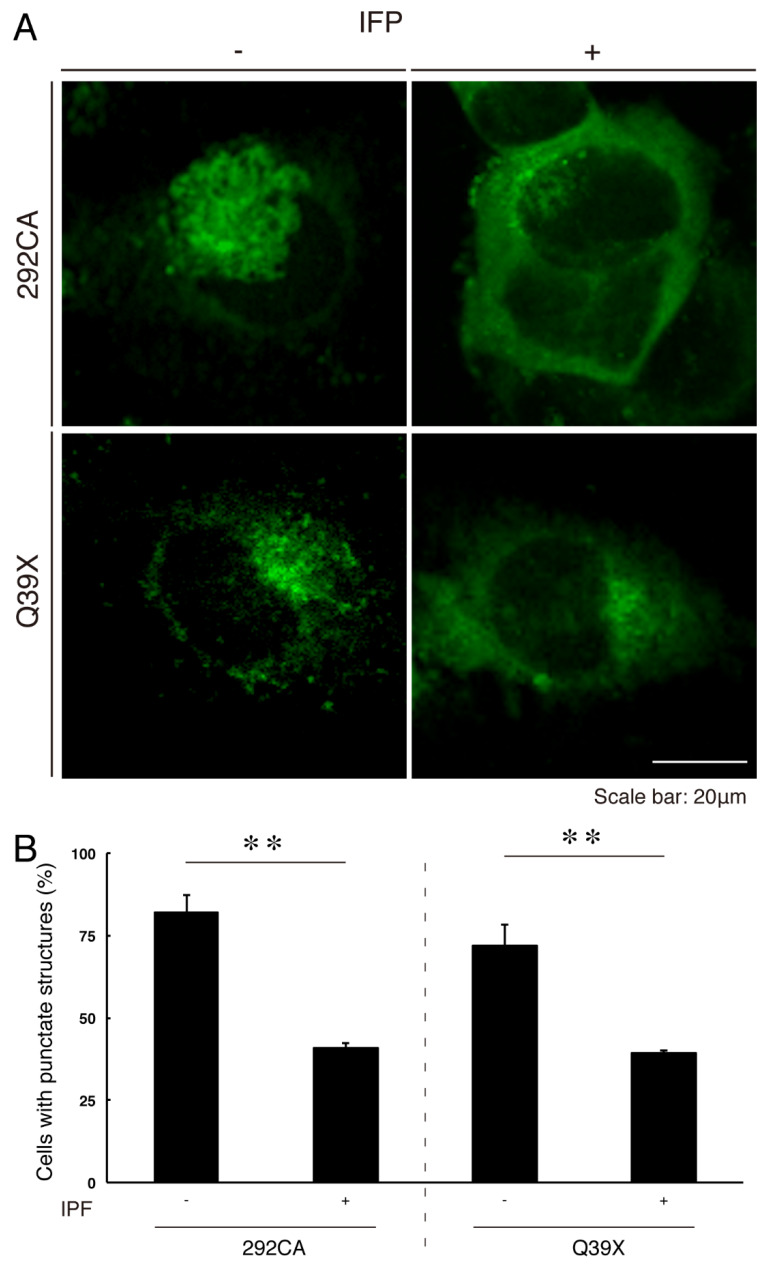
Ibuprofen decreases aggregate-like punctate structures of the AIMP1′s 292CA or Q39X mutant proteins. (**A**) N1E-115 cells were transfected with the plasmid encoding the 292CA or Q39X mutant constructs of AIMP1 with a GFP-tag in the presence or absence (vehicle control) of ibuprofen (IPF). Representative fluorescence images (green) are shown. The scale bar indicates 20 μm. (**B**) The percentages of cells with punctate structures are shown in a graph (**, *p* < 0.01; n = 3 fields [total 240 cells]).

We tested the effects of ibuprofen, a medication in the nonsteroidal anti-inflammatory drug (NSAID) class that possesses protective properties for neuronal cells [19,20,21], on inhibitory differentiation in N1E-115 cells harboring the 292CA or Q39X mutant constructs. Treatment of these cells with ibuprofen for 2 or 3 days reversed inhibitory differentiation into phenotypes by more than 75% or more than 80%, respectively (Figure 12A,B). Similar results were obtained in cells harboring the Q39X mutant constructs (Figure 12C,D). Further, while the 292CA or Q39X mutant proteins exhibited aggregate-like structures in more than 75% of cells, treatment with ibuprofen decreased this incidence to less than 50% (Figure 13A,B). Consistently, ibuprofen diminished the presence of actin and 292CA mutant proteins in the lysosome (Figure 9D, blots 1 and 3). A similar change to decrease in localization in the lysosome was observed in the case of Q39X mutant proteins (Figure 9D, blot 1). Together, these results suggest that ibuprofen reverses the mutant-mediated phenotypes.

## 4. Discussion

Previous studies on PMD have illustrated that hypomyelination is due to unfolded protein response (UPR) in the ER in oligodendrocytes. PMD is associated with mutations of the *plp1* gene, whose products are the major myelin membrane proteins. Various types of mutations are thought to lead to the accumulation of unfolded, aggregated PLP1 proteins in the ER in oligodendrocytes, triggering UPR [5,6,7,8]. UPR generally results in a shift in protein effect from triggering the cellular survival machine to irrevocably committing to the pathway to cell death. These mutations are thus toxic gain-of-function mutations. The mutation responsible for HLD2 disrupts the folding of the gap junctional GJC2 proteins, which accumulate as aggregated proteins in the ER in oligodendrocytes and in turn initiate UPR [9]. The gene affected by HLD5 normally encodes FAM126A (also called HYCCIN or DRCTNNB1A), a subunit of the phosphatidylinositol 4-kinase enzyme [22]; the missense mutation leads to ER localization of this enzyme in oligodendrocytes as unfolded, aggregated FAM126A proteins, triggering UPR [23], although it remains unclear how cytoplasmic proteins are transported within the ER as aggregated proteins [24]. Since the aggregation of GJC2 or FAM126A proteins leads to their accumulation in the ER, it is likely that the mutations in HLD2 or HLD5 are toxic gain-of-function mutations. Some HLD-associated mutations may cause aggregation of cognate proteins, accumulating them in the ER and thereby triggering UPR [5,6,7,8].

While PLP1 and GJC2 are highly expressed in oligodendrocytes but not in neuronal cells [5,6,7,8], FAM126A is widely expressed throughout a variety of tissues [23]. Indeed, the aggregation of mutated PLP1 or GJC2 proteins is a phenomenon specific to oligodendrocytes, whereas the effect of aggregation of mutated FAM126A proteins seems unlikely to be specific to oligodendrocytes. It is already known that the HLD5-associated mutation of FAM126A causes congenital cataracts [23]. This reminds us of the possibility that aggregation of AIMP1, which is widely expressed in tissues, can cause pathological effects not only in oligodendrocytes but also in various other types of cells including neuronal cells. HLD3-associated AIMP1 mutations are actually associated with severe neurological disorders in which neuronal cells are strongly damaged [10]. Thus, we investigated whether the expression of mutated AIMP1 proteins has cellular pathological effects on neuronal cells. In the present study, we have found that, in N1E-115 cells, the expression of mutated AIMP1 proteins, but not that of wild type proteins, leads to protein accumulation primarily in the lysosome, which inhibits cell differentiation. If mutant proteins are accumulated in the lysosome, it is presumed to be degraded; but mutants are indeed present in the lysosome. The reason may be simply due to blockage of lysosomal degradation system by mutant proteins. Alternatively, the lysosomal system may be overloaded and can no longer be functional for degradation. In either case, it is likely that accumulation of mutant proteins in the lysosome is responsible for inhibitory differentiation in N1E-115 cells. These findings are fairly consistent with some of the pathological effects of mutations of AIMP1 on neuronal tissues [10,12,13]. From this perspective, HLD3-associated AIMP1 mutations may also be toxic gain-of-function mutations. Mutant proteins do not seem to cause cell death, since trypan-blue-incorporating cells are likely comparable in cells expressing wild type or mutant proteins. It is noteworthy that cellular phenotypes induced by HLD3-associated AIMP1 mutants are reversed by ibuprofen, which has neuroprotective effects with neuronal process-promoting activities but unlikely promotes cell survival [19,20,21].

AIMP1 participates in forming the multiple tRNA synthetase complex, functioning as an adaptor protein modulating the aminoacylation activities of tRNA synthetases in the cytoplasm [13,14]. This multiple complex contains other adaptor proteins such as AIMP2, AIMP3, and some tRNA synthetases. It is therefore thought that AIMP1 acts together with AIMP2 and AIMP3 to induce the full aminoacylation activity. Since HLD3-associated mutations cause an early premature cessation of AIMP1 translation, mutant proteins either lack or have decreased adapter activity of the multiple tRNA synthetase complex [13,14]. From this point of view, HLD3-associated mutations are loss-of-function mutations. The loss or decreased activity of adaptor proteins contained in the multiple tRNA synthetase complex is confirmed by the recent report that HLD17-associated AIMP2 mutation causes early premature cessation of AIMP2 translation, although HLD17-affected cells are most likely to be oligodendrocytes rather than neuronal cells [25]. Further studies on genes encoding adaptor proteins related to the multiple tRNA synthetase complex may identify disease-associated missense mutations other than premature stop mutations whose amino acid positions are essential for their adaptor activities. Such analyses will allow us to understand whether AIMP1 mutations cause HLD3 phenotypes primarily through loss of AIMP1 activity, protein aggregations, or both.

AIMP1 is also known as an endothelial monocyte activating polypeptide II (EMAPII) precursor protein p43. The C-terminal half of AIMP1 (EMAPII) is secreted as a soluble ligand from some types of cells including immune cells; this ligand then functions as an inflammatory cytokine [26]. Additionally, EMAPII is involved in the regulation of inflammation and angiogenesis. EMAPII specifically binds to cell surface receptor CD23 and mediates further secretion of various inflammatory cytokines [26], although CD23 was originally identified as a B-cell specific antigen that was also a low-affinity receptor for IgE. However, CD23 is probably absent or expressed at low levels in the CNS. It is possible that the AIMP1 or EMAPII receptor in the CNS is a protein or proteins other than CD23. Since immature HLD3-associated AIMP1 mutant proteins are deficient in the C-terminal EMAPII region of AIMP1, complete loss of its ligand activity may be associated with the molecular and cellular pathologies of HLD3 and/or their promotion.

**Figure 14:**
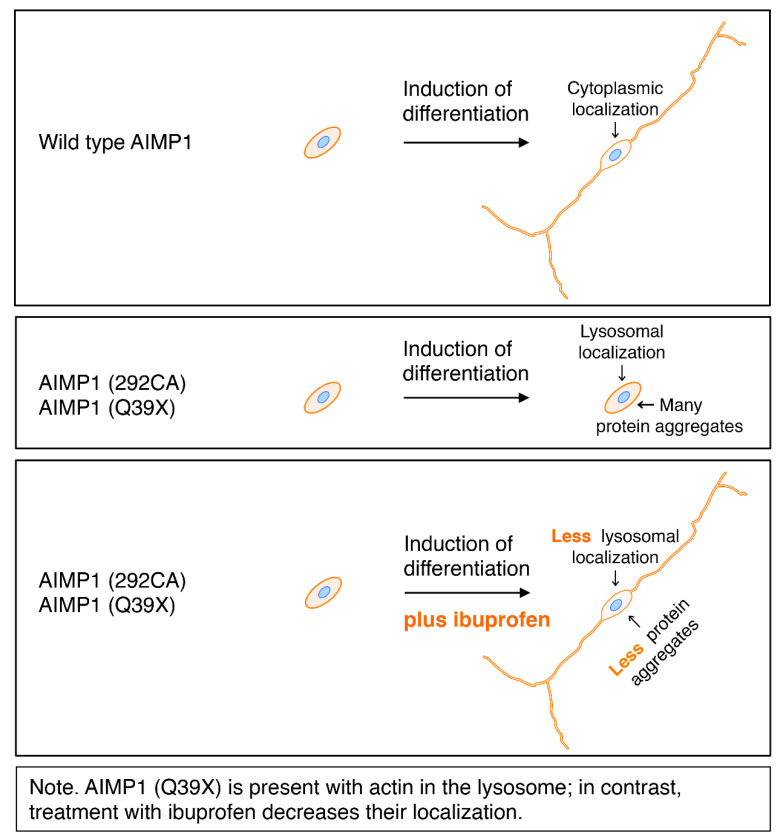
Schematic diagram of effects of HLD3-associated mutants on cell morphological changes in N1E-115 cells.

Herein, we demonstrate that the HLD3-associated mutations (i.e., the 292CA and Q39X mutations) of cytoplasmic AIMP1 proteins cause them to localize primarily in the lysosome (Figure 14). The 292CA mutation, whose product displays a frame-shift-type harboring two-base (CA) deletion at the 292nd nucleotide position, generates the 292CA mutant-specific de novo polypeptide. Since the peptide contains basic amino acids, it is conceivable that it physically binds to acidic proteins such as actin. Despite the normal localization of actin in the lysates of no transfected cells, ectopic expression of the 292CA mutant proteins results in detection of actin proteins together with 292CA proteins in the lysosome fraction. Further studies will improve our understanding of how HLD3-associated AIMP1 mutant proteins including Q39X mutant proteins are accumulated in the lysosome to inhibit morphological differentiation with disorganized actin networks. Some of mutant proteins are also accumulated in the Golgi body. It will be important to clarify how cytoplasmic AIMP1 proteins are transported within the lysosome and/through the Golgi body as aggregated proteins by HLD3 mutations. Additionally, clarification of the detailed mechanisms underlying the effects of ibuprofen may elucidate not only how HLD3-associated cellular phenotypes are reversed but also whether other compounds could be used for this purpose both in vivo as well as in pathological cells. These future studies may lead to the development of a drug-target-specific medicine for the treatment of HLD3 and other possible AIMP1-containing multiple tRNA synthetase complex-related diseases [10,11,12,13].

## Supplementary Materials

Supplementary Materials are available online at https://www.mdpi.com/2305-6320/7/5/25/s1. Figure S1: Full gel images in the Figure 9D. Cropped images are indicated by black squares.
