# Supplementary material for: Rare Neurologic Disease-Associated Mutations of AIMP1 Are Related with Inhibitory Neuronal Differentiation Which Is Reversed by Ibuprofen
Source: Medicines (Basel). 2020 May 6;7(5):25. doi: 10.3390/medicines7050025 (PMC7281511; doi:10.3390/medicines7050025)
Supplement: Supplementary file 1 [file medicines-07-00025-s001.pdf]

Supplementary Materials: Rare Neurologic Disease-Associated Mutations of AIMP1 are Related with Inhibitory Neuronal Differentiation Which is Reversed by Ibuprofen

Yu Takeuchi, Marina Tanaka, Nanako Okura, Yasuyuki Fukui, Ko Noguchi, Yoshihiro Hayashi, Tomohiro Torii, Hiroaki Ooizumi, Katsuya Ohbuchi, Kazushige Mizoguchi, Yuki Miyamoto and Junji Yamauchi

Figure S1

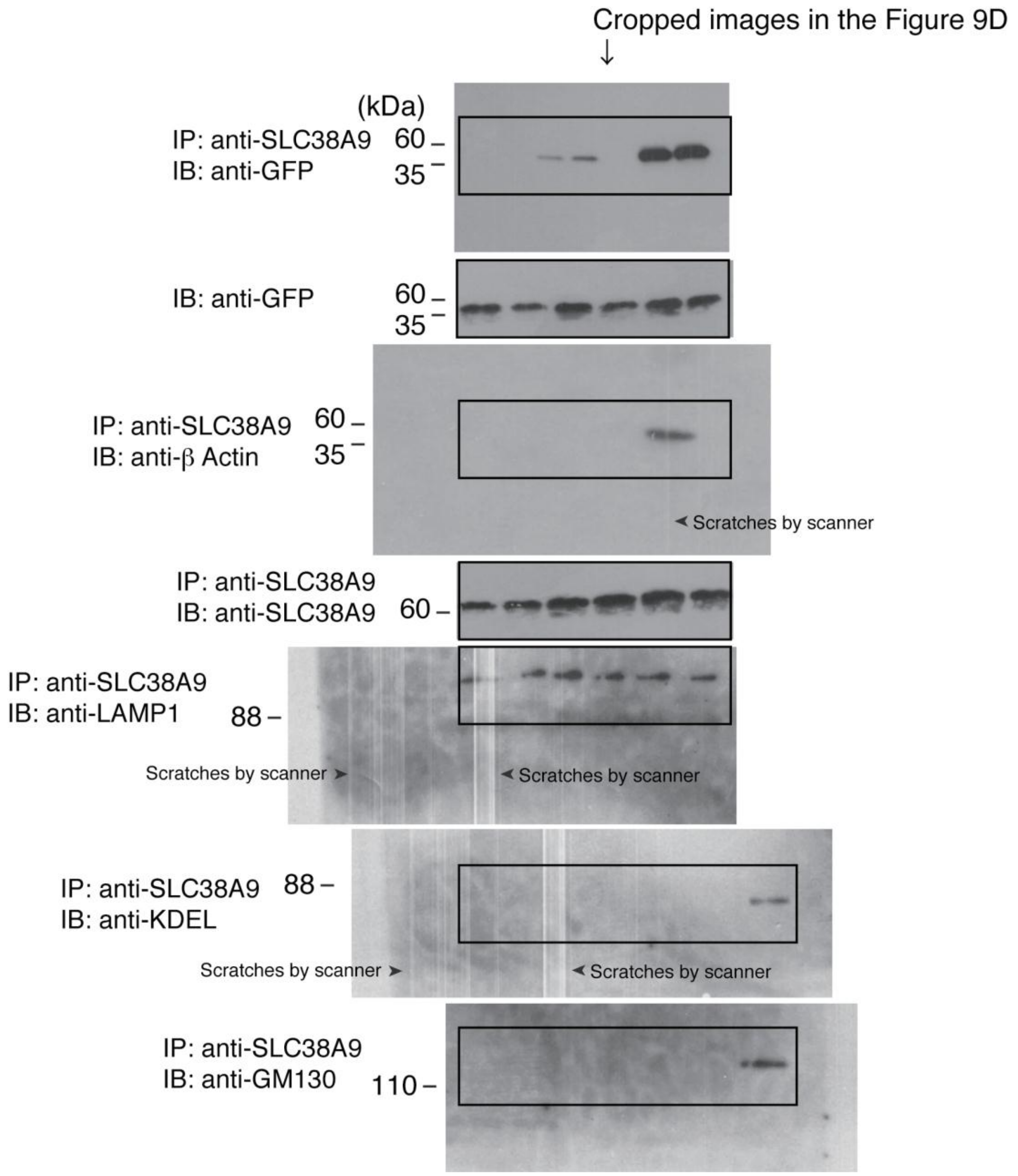

Figure S1. Full gel images in the Figure 9D. Cropped images are indicated by black squares.
